# Abla Mehio Sibai: championing healthy ageing in Lebanon

**DOI:** 10.2471/BLT.23.030923

**Published:** 2023-09-01

## Abstract

Professor Abla Mehio Sibai talks to Lynn Eaton about the need for cross-disciplinary approaches to supporting healthy ageing and the challenges posed by the collapsing public sector in Lebanon.

*Q: What drew you to focus on the health of older people in Lebanon?*

A: I graduated in pharmacy in 1977 and went straight into the profession I had trained for, but Lebanon’s atrocious civil war that ran from 1975 to 1990 changed my outlook. Witnessing the impact of the war on relatives and friends, and especially on vulnerable older people, made a big impression on me. My father was among those severely affected, losing a vibrant business in the prime of his productive years, being forced into early retirement, and eventually becoming ill. Because he was self-employed, he had no health coverage and no pension. His situation reflected broader challenges for the older Lebanese population that included huge inequities in pensions, and absurdities such as people covered by the National Social Security Fund losing their health insurance upon retirement.

Having been exposed to all that, I decided to immerse myself in public health issues. I pursued a PhD in epidemiology and, in my dissertation, researched the impact of the war on vulnerable older people in terms of morbidity and mortality. It was harrowing in some ways but fascinating too, each study unearthing new questions. For example, I remember my very first publication on the association between war-related stressors and coronary artery disease leading to a subsequent investigation that revealed the alarming levels of unindicated cardiac catheterization in Lebanese hospitals. The data produced from this study informed stricter guidelines for cardiac catheterization imposed by the Ministry of Public Health, and set the stage for establishing the national Lebanese Interventional Coronary Registry, which serves to monitor and evaluate catheterization use and to compare clinical practices at the national and international level. I also examined the gaps in routine sources of data in the country and researched the reliability and validity of death certification. I later set out to capture the broader demographic and epidemiological transitions taking place in Lebanon, producing the first national report on older adults in 1998 and, in 2001, I co-led the country’s first ‘Global Burden of Disease’ project. That study set the stage for another nationwide survey of noncommunicable disease and risk factors.

“Lebanon’s atrocious civil war […] changed my outlook.”

*Q: When did your focus shift to broader social and structural issues impacting older people’s health?*

A: It grew out of the work I did on chronic disease. It became clear to me that the well-being of older persons extends far beyond biology and disease. Consequently, I shifted my research focus towards comprehending the intricate connections between psychological experiences, including predictable and unpredictable life transitions, and the social and the physical built environment in shaping our health as we grow older. I researched the factors that drive older adults to self-rate their health as 'good or excellent', despite functional limitations or a lack of individual, social or economic resources, and also examined the impact of early-life displacements on a group of disadvantaged, ethnically diverse older people. That work involved an effort to characterize their socio-economic context and relevant geo-historical factors, which entailed the application of a more cross-disciplinary perspective.

*Q: You created the Center for Studies on Ageing in 2008 – to what extent does it encourage cross-disciplinary approaches?*

A: The Center was a deliberate effort to ‘walk the cross-disciplinary talk’. To create it I basically had to move out of my health research comfort zone, establishing a virtual platform committed to translating research on older adults into policies and programmes that bring together academics, service providers, heads of nongovernmental organizations, leaders, students and older adults in roundtable debates, conferences and workshops. The Center has yielded a range of policy briefs and country reports and also acts as a repository, archiving research on ageing in Lebanon and the Arab world. It has also played an important role in supporting the development of national, regional and international collaborations. For example, in 2014, we became a network member of HelpAge International, an international nongovernmental organization that helps older people meet challenges ranging from discrimination to poverty.

*Q: In 2010 you co-founded the University for Seniors at the American University of Beirut. Can you tell us about that?*

A: The university is a pioneering late-life learning programme made for and with older adults. The programme provides opportunities for seniors to stay intellectually engaged and socially connected. Because it is hosted within the university, senior students rub shoulders with younger students, benefiting both generations. We started small, but now offer more than 100 lectures, courses, and workshops, and enrol nearly 500 participants each year. The programme offers the strongest in-the-field evidence of the value of intellectual stimulation and social interaction in delaying cognitive and physical decline in older people.

*Q: Do you have plans to expand the programme?*

A: To date, the programme remains the only one of its kind in the Arab region, but during the COVID-19 pandemic we went online and witnessed significant participation outside the country, from Türkiye, Egypt, the United Arab Emirates, and even people in the United States of America. This digital expansion also promoted greater inclusion, increasing access to Arabic-speaking participants who are challenged by physical limitations or geographic location. The University for Seniors has been cited as a case study in several international reports, and been selected as one of the 10 most innovative social interventions for older persons in low- and middle-income countries globally. Such recognition is very encouraging and fuels our hope for greater expansion and impact in the future.

*Q: You have also led three mapping reviews of policies and programmes in the Eastern Mediterranean Region and the development of the National Strategy for Older Persons in Lebanon. To what extent has that work fed through into government policy?*

A: The regional reviews and the national strategy are only first steps towards identifying priority areas for action and evidence-based policy formulation; but with no political commitment, feasible action plans with targets, timelines and resources, there will be no implementation. Unfortunately, older people in Lebanon have historically been marginalized in the implementation of policies and programmes. With the ongoing financial crisis which started in 2019, including a total collapse of the public sector and retirement savings being eroded by the banks, older people are at higher risk of poverty and exclusion than ever. At the same time, the proportion of older people is growing.

“Older people are at higher risk of poverty and exclusion than ever.”

*Q: What proportion of the Lebanese population is over 65?*

A: No recent data exist, but in 2020 it was estimated that around 11% of the population of 6 million people were over the age of 65. This is by far the highest among Arab countries. With increasing emigration of young adults, and an influx of retired older adults who had been working in the Gulf States where there is no prospect of naturalization, that percentage is likely to have increased. It is also important to note that life expectancy in Lebanon is relatively high at 79 years for men and 82 for women. These demographic trends, and the increasing prevalence of chronic and disabling degenerative diseases among older people, is putting tremendous pressure on the health and social care system.

*Q: How is the demand for care being met?*

A: It is not. The ongoing financial and economic crisis in Lebanon, described by the World Bank as one of the worst in recent history, is straining the already limited ability of the government to provide adequate social protection and health-care coverage. It is estimated that public funds can now only cover 10% of medical costs. As a result, older individuals are being pushed into catastrophic out-of-pocket expenditures and relying on family support. The caregiving landscape in the country remains largely unexamined, but evidence suggests that care for older disabled people is provided by their families. Female family members almost always fill the care gap, with many providing care simultaneously to both children and older family members. In the past, migrant workers used to be a source of paid informal care for older individuals in Lebanon. However, due to the depreciation of the Lebanese currency and increased poverty among once middle-class older adults, this is no longer a viable option. The situation is all the more precarious in the refugee population. Lebanon currently hosts the largest number of refugees per capita worldwide, with an estimated 1.5 million Syrian refugees and around 250 000 people of other nationalities. The collapse of the economy, along with the impact of the COVID-19 pandemic and Beirut blast have only exacerbated the challenges they face. These include extreme vulnerability relating to the sheer length of time they have been in Lebanon. Refugees are ageing here, and many are facing a syndemic of noncommunicable diseases and mental health issues. Medication shortages, and a lack of palliative care for those with life-limiting conditions, pose significant challenges that humanitarian agencies struggle to meet. Interventions to improve the care provided to refugees must be integrated into the overall health-care system of the country, without segregation between refugees and the host communities.

*Q: What plans do you have for the future?*

A: We have a number of exciting projects lined up at AUB. For example, we recently secured a 5-year grant from the National Institutes of Health in the United States of America to study the connection between learning and cognitive health, which will focus on seniors attending the University for Seniors. I am enthusiastic about the possibilities it will unlock, not only for future research questions but also for showcasing the impact of the University for Seniors, making it an attractive model to be emulated in other countries and in low-resource settings. Ultimately, my vision is to establish a Programme of Excellence on Ageing at our university—an ambitious initiative that would bring together various disciplines and foster interdisciplinary dialogue, driving research, education, policy development and advocacy on ageing issues. I am deeply passionate about this project and hope that funding agencies will respond to my call to actively support it.

